# Fungal infections of the central nervous system in HIV-negative patients: Experience from a tertiary referral center of South India

**DOI:** 10.4103/0972-2327.64635

**Published:** 2010

**Authors:** K. N. Ramesha, Mahesh P. Kate, Chandrasekhar Kesavadas, V. V. Radhakrishnan, S. Nair, Sanjeev V. Thomas

**Affiliations:** Department of Neurology, Sree Chitra Tirunal Institute for Medical Sciences and Technology, Trivandrum-695 011, India; 1 Department of Neuroradiology, Sree Chitra Tirunal Institute for Medical Sciences and Technology, Trivandrum-695 011, India; 2 Department of Neuropatholgy, Sree Chitra Tirunal Institute for Medical Sciences and Technology, Trivandrum-695 011, India; 3 Department of Neurosurgery, Sree Chitra Tirunal Institute for Medical Sciences and Technology, Trivandrum-695 011, India

**Keywords:** Amphotericin, central nervous system mycosis, outcome

## Abstract

**Objective:**

To describe the clinical, radiological, and cerebrovascular fluid (CSF) findings and the outcome of microbiologically or histopathologically proven fungal infections of the central nervous system (CNS) in HIV-negative patients.

**Methodology and Results:**

We identified definite cases of CNS mycosis by screening the medical records of our institute for the period 2000–2008. The clinical and imaging details and the outcome were abstracted from the medical records and entered in a structured proforma. There were 12 patients with CNS mycosis (i.e., 2.7% of all CNS infections treated in this hospital); six (50%) had cryptococcal infection, three (25%) had mucormycosis, and two had unclassified fungal infection. Four (33%) of them had diabetes as a predisposing factor. The common presentations were meningoencephalitis (58%) and polycranial neuritis (41%). Magnetic resonance imaging revealed hydrocephalus in 41% and meningeal enhancement in 25%, as well as some unusual findings such as subdural hematoma in the bulbocervical region, carpeting lesion of the base of the skull, and enhancing lesion in the cerebellopontine angle. The CSF showed pleocytosis (66%), hypoglycorrhachia (83%), and elevated protein levels (100%). The diagnosis was confirmed by meningocortical biopsy (in three cases), paranasal sinus biopsy (in four cases), CSF culture (in three cases), India ink preparation (in four cases), or by cryptococcal polysaccharide antigen test (in three cases). Out of the ten patients for whom follow-up details were available, six patients recovered with antifungal medications (amphotericin B, 1 mg/kg/day for the minimum period of 6 weeks) and/or surgical treatment. Four patients expired (only one of them had received antifungal therapy).

**Conclusions:**

Most patients with CNS mycosis recover with appropriate therapy, but the diagnosis and management of these rare infections remains a challenge to clinicians.

## Introduction

Fungal infections of the central nervous system (CNS) are relatively uncommon neuroinfections that are increasingly being recognized these days. This is mainly due to the increased awareness of clinicians about these conditions, the growing pool of immunocompromised host, the advances in imaging, and the availability of microbiological techniques to confirm the diagnosis from body fluids and specimens.[1,2] CNS mycosis can present as meningitis, encephalitis, stroke, intracranial mass lesions, spinal cord syndromes, or base of skull disorders.[2,3] The important conditions that predispose to CNS mycosis include AIDS, diabetes mellitus, use of immunosuppressants, organ transplantation, and hematological malignancies.[1-4] Though mortality and morbidity from CNS mycosis has improved over the decades, it remains high.[1,5] Against this background, we aimed to analyze the spectrum of CNS mycosis seen in HIV-negative patients treated in our institute over the last 8 years and to examine the outcome in these cases.

## Materials and Methods

This retrospective study was carried out at Sree Chitra Tirunal Institute for Medical Sciences and Technology, Trivandrum, a tertiary referral center for neurological and cardiac disorders. We defined 'CNS mycosis' as an infection of the central nervous system in a patient admitted with a neurological disorder, with the fungal etiology having been confirmed by histopathological or microbiological methods. We screened the case records of all admissions to the neurology services from January 2000 to December 2007 to identify all cases of CNS mycosis. During this period there were a total of 453 cases with neuroinfections and 12 (2.7%) of them satisfied our criteria for CNS mycosis. Their demographic data, clinical features, imaging findings, cerebrospinal fluid (CSF) picture, treatment details, and outcome were abstracted from the clinical records and entered into a structured proforma. For follow-up, patients and their caregivers were contacted either by telephone or by mail for details of current health status.

## Results

Twelve patients (six males and six females) with confirmed CNS mycosis were treated at our institute during the study period. Their mean age at presentation was 41.2 years (range 23–66 years). The mean duration of symptoms at presentation was 73 days (range 13–120 days). The various etiological agents were cryptococcus (six patients), mucormycosis (three), and aspergillus fumigatus (one). In the remaining two cases, the type of fungi could not be identified. Diabetes mellitus and long-term use of steroid for treatment of autoimmune disorders were the predisposing factors in four and two cases, respectively. Infection occurred in the setting of head injury and post neurosurgical procedure in one patient each. Four patients had no identifiable risk factors.

Patients presented with one of the two broad clinical presentations:

- Meningitis syndrome: Seven patients had meningitis syndrome with fever and symptoms and signs of raised intracranial pressure and meningeal irritation. Two of them had hemiplegia due to vascular involvement and one had multiple cranial nerve palsies. Five had cryptococcal meningitis and one had postoperative meningitis due to aspergillus fumigatus.
- Cranial neuropathy syndromes: Five patients had polycranial neuritis, three with ocular motor involvements and two with lower cranial nerve palsies. Two each had cryptococcoma and mucormycosis.

**Figure 1:**
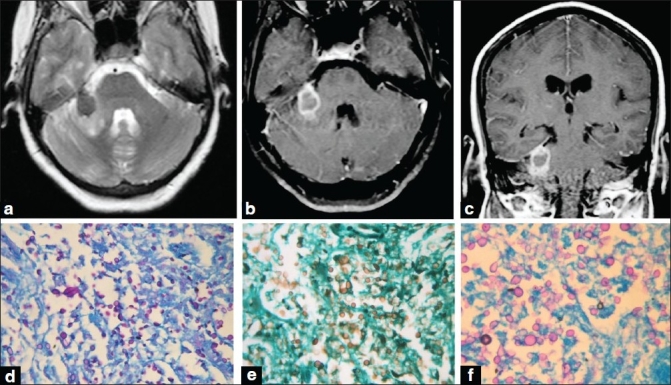
A 52-year-old lady presented with insidious onset, gradually progressive, right-sided trigeminal and facial nerve palsy of 4 months' duration. (a) Magnetic resonance imaging T2W axial sequence shows heterogeneously hyperintense lesion; (b, c) postcontrast T1W axial and coronal sequence shows enhancement of the lesion (d) in the right cerebellopontine angle. The histopathology of the postoperative surgical specimen by Grocott-Gomori stain, (e) periodic acid-Schiff stain, and PAS with Alcian blue stain (f) were positive for cryptococcus. CSF culture and India ink preparation were also positive for cryptococcus. The patient made a near total recovery with amphotericin and fl uconazole, though she had residual hydrocephalus, which improved with a ventriculoperitoneal shunt.

Routine hematological workup was unremarkable for most patients. Seven patients had leukocytosis, with a mean cell count of 18,857 cells/mm^3^ (range: 12,500–32,000 cells/mm^3^), and nine had raised erythrocyte sedimentation rate of more than 50 mm in 1 h. MRI of the brain showed hydrocephalus in five patients and meningeal enhancement in three. Findings suggestive of choroid plexitis and vascular invasion of the middle cerebral artery was seen in one patient each. Other MRI findings included a carpeting lesion of the base of skull, an enhancing lesion in the cerebellopontine angle [Figure 1], and a subdural hematoma located in the bulbocervical location (this patient presented with spastic quadriparesis).

All patients had undergone lumbar puncture for cerebrospinal fluid (CSF) study after cerebral imaging. The mean cell count in the CSF was 377 mm^3^ (range 5–1100 cells/mm^3^), eight had increased CSF cell count; only two had predominant polymorphonuclear leukocytosis. The mean CSF protein was 103.75 gm% (range 44–183 gm%) and mean CSF sugar was 33.91 mg% (range 3–65 mg%). Four patients had hypoglycorrhachia of less than 20% of the corresponding blood sugar. Cryptococcus was identified in the CSF by India ink preparation and was grown from the CSF in three patients. CSF smear was positive by PAS stain for mucor in one patient. The paranasal sinus biopsy was positive for a specific fungus in three patients and unclassified in one. Cryptococcal polysaccharide antigen by latex agglutination method was positive in three out of the four patients in whom it was done. Meningocortical biopsy confirmed a specific fungus in three patients (cryptococcus, mucor, and *Aspergillus fumigatus* in one patient each). The workup carried out for bacterial meningitis and other chronic meningitides included CSF Gram's stain, polymerase chain reaction for *Mycobacterium tuberculosis*, cultures, and cytology for malignant cells; these tests were negative in all cases.

Seven patients were treated with 1 mg/kg/day of intravenous amphotericin B either for 6 weeks or till two sequential CSF cultures were negative (i.e., those that had been positive earlier). In addition, flucytosine at dose of 100 mg/kg/day for 2 weeks and fluconazole 400 mg/day for 8 weeks were given in one case each. Reversible renal failure and hepatorenal syndrome were noted in one patient each as complications of amphotericin treatment. Three patients underwent paranasal sinus debridement, two underwent resection of the intracranial/spinal mass, and one required ventriculoperitoneal shunt for hydrocephalus.

Follow-up data were available in ten cases; the mean duration of follow-up was 2.5 years. Five patients became asymptomatic and one made an incomplete recovery, with hydrocephalus as a sequel. Four patients expired (40%) in the hospital [hospital stay ranged from 3–36 days (mean 13 days)]. Three patients had cryptococcal infection and one had mucormycosis. Only one of the four patient who expired had received antifungal therapy. None of the surviving six patients had recurrence after the mean follow-up of 2.5 years. The clinicoradiological features and outcomes are summarized in Table 1.

**Table 1:** Clinical features, investigation findings, and outcome of the study cohort

Age/sex	Neurological syndrome	Predisposing factor	CSF cells/mm^3^	CSF protein (gm%)	CSF sugar (mg %)	Imaging	Confirmatory test	Organism	Treatment	Outcome
23/M	Polyneuritis cranialis	Nil	350	152	62	Carpeting lesion in the base of the skull	CSF -Antigen	C	Amph, Flucytosine	Asymp
30/M	Meningitis	Diabetes mellitus	1110	183	17	Dilated V-R space in basal ganglia, hydrocephalus, infarct	CSF-culture, India ink	C	Amph	E
52/F	Meningitis, multiple CN palsy	Animal husbandry employee	740	90	10	CP angle enhancing lesion	CSF ‐ culture, India ink, antigen, histopathology	C	Amph, fluconazole	Incomplete recovery, hydrocephalus
57/M	Meningitis, seizures	Myasthenia gravis on steroid	2	95	8	Hydrocephalus, infarct	India ink, antigen	C	Nil	E
30/M	Meningitis, multiple CN palsy, hemiplegia	Nil	630	126	3	Infarct	India ink	C	Nil	E
34/F	Meningitis	SLE on steroid, AZA	80	108	45	Choroid plexitis, hydrocephalus	Culture	C	Amph	Asymp
31/M	High cervical myelopathy	Post-traumatic	2	133	30	Bulbo-cervical SDH, meningeal enhancement, hydrocephalus	Histopathology	M	NA	NA
60/M	Cavernous sinus syndrome, ICA infarct	DKA	620	97	46	Cavernous sinus ICA occlusion, infarct, hydrocephalus	PNS biopsy	M	Nil	E
34/M	Orbital apex syndrome, hemiplegia	Nil	5	54	67	Orbital apex lesion, MCA infarct	PNS biopsy	M	Amph	Asymp
25/F	Meningitis, hemiplegia, seizures	Post op colloid cyst resection	200	77	40	Meningeal enhancement, hydrocephalus	Histopathology	Asp	Amph	Asymp
66/F	Bilateral cavernous sinus syndrome	Diabetic nephropathy	5	86	45	Meningeal and cavernous sinus enhancement,	PNS biopsy	U	NA	NA
60/F	Meningitis	Poorly controlled diabetes	800	44	34	Sphenoidal sinus mass with fluid level	PNS biopsy	U	Amph	Asymp

M = Male, F = Female, AZA: Azathioprine, DKA: Diabetic ketoacidosis, C = Cryptococcus, M - Mucormycosis, Asp = Aspergillus fumigatus, U = Unclassifi ed fungus, E = Expired, NA = Not available, Asymp = Asymptomatic, Amph = Amphotericin, PNS = Paranasal sinuses

### Subgroup analysis

The majority of those with the meningitis syndrome (*n* = 7) had cryptococcus infection (71%). In those with the cranial neuropathy syndromes (*n* = 5), ocular motor involvement was seen only with mucormycosis (*n = 2*) and bulbar involvement/ lower cranial palsy was seen only with cryptococcus infection (*n* = 2). There was no statistically significant difference between those who survived (*n = 6*) and those who died (*n* = 4) with regard to age at presentation, presence of predisposing disorders, duration of symptoms, and specific MRI findings. However, only one of the four patients who died received any antifungal therapy, whereas all surviving patients had received antifungal therapy.

## Discussion

In this retrospective analysis, we have presented the clinicoradiological and CSF features and outcome of 12 consecutive patients with CNS mycosis. Fungal infection of the CNS was relatively rare in our experience (2.7% of all CNS infections). In other series that included patients with HIV and other immunocompromised patients, higher frequency of fungal meningitis (3–30% of all chronic meningitis) have been reported.[5,6] The risk factors for CNS mycosis in this series included diabetes mellitus, long-term steroid therapy, neurosurgery, and trauma. Other risk factors mentioned in the literature like HIV infection, organ transplantation, malignancy, etc. were not seen in our study cohort. This difference may be due to geographic and institutional referral bias. Cryptococcal meningitis is the most common clinical fungal infection of the CNS.[1,6,7] Certain syndromes are more common with some particular fungal type and this may guide further investigation and treatment. Signs of meningeal irritation occurred in 86% of cryptococcus infections, whereas focal neurological deficits were seen 50% of patients with aspergillosis in a series consisting of 57 autopsied patients with CNS fungal infections.[7] There are certain differences noted between immunocompetent and immunocompromised hosts in the clinical presentation: the duration of symptoms is likely to be longer and the incidence of neck rigidity is higher in immunocompetent individuals.[6]

Our patients presented with one of two broad syndromes: meningoencephalitis or cranial neuropathy. There was a distinct association between the fungal etiology and presentation. The majority of patients with cryptococcal infections presented with meningitis; two patients also had cryptococcoma. The other rare CNS manifestations described in cryptococcal infection are subdural effusion, dementia, and ischemic strokes, none of which were seen in our cohort.

We found lymphocytic pleocytosis in 6 patients (50%) and polymorphonuclear pleocytosis in two – one in a cryptococcus infection and the other in an aspergillus infection. In the literature, polymorphonuclear pleocytosis said to be common in aspergillus and candida infection.[8] Immunocompromised patients, particularly those with HIV infection and AIDS, have higher a fungal load and tend to have higher yield with microbiological studies. In some series, India ink test was positive for cryptococcus in 80% cases, fungus culture was positive in 90%, cryptococcal polysaccharide antigen test (by latex agglutination or ELISA method) was positive in 90% cases.[8-10]

The radiological features of CNS mycosis tend to be nonspecific. They include hemorrhagic/edematous lesions, solid enhancing lesion, abscess, infarction, mycotic aneurysms, meningeal enhancement, hydrocephalus, sinusitis, etc.[11,12] MR imaging features in cryptococcal infections are cryptococcoma in basal ganglia and midbrain, hydrocephalus, and meningeal enhancement.[11,12]

Amphotericin B has poor penetration of the meninges, though it is a potent antifungal agent. Hence, it is often combined with flucytosine and fluconazole, which have better CSF penetration.[4] The mean treatment duration in those patients who completed treatment was 6.3 weeks. The recommended duration of treatment is 6–12 weeks or till culture becomes negative. The most important treatment-limiting toxicity reported with amphotericin is renal failure. This complication was seen in two out of seven of our patients, but the changes were reversible. Continuous infusion of conventional amphotericin is much cheaper and is probably as safe as treatment with the lipid-based formulation.[4]

Fungal infections of CNS often require surgical intervention for diagnosis (stereotactic biopsy) and therapy (CSF shunt procedures for hydrocephalus and resection of the fungal abscess or granuloma).[13] In our series, three patients underwent paranasal sinus debridement, two underwent resection of the intracranial/spinal mass, and one required a ventriculoperitoneal shunt for hydrocephalus. Debridement of sinuses is necessary in all forms of rhino-orbito-cerebral mucormycosis.[14-17] It is important to carefully manage the predisposing conditions like diabetes while the patient is undergoing antifungal medications and surgery. Patients with AIDS require highly-active antiretroviral therapy (HAART) and long-term fluconazole maintenance therapy.[17]

The overall survival rate in this study was 60%, whereas the survival rate for those who received treatment was 86% (six out of seven patients). According to published literature, the survival rate varies between 40% and 92%.[1-4,6,7] In a study of 129 cases, the overall survival rate was 72%.[1] It was higher for cryptococcal and candida infection than for aspergillosis. In general, immunocompetent individuals with early diagnosis and treatment had better outcomes.[18] In a study of 38 patients with rhinocerebral mycosis, 47% died; the predictors of mortality were elderly age, intracranial extension, and immunocompromised state.[15] In view of the small number of cases in our study, we could not ascertain the prognostic factors.

In our small series, there were no specific clinical or imaging parameter that predicted higher mortality or complications. We observed that fungal etiology can be identified by a diligent search for fungi in the CSF by routine mycological tests in most cases. Stereotactic biopsy or biopsy of the any granuloma present in the paranasal sinuses are likely to provide accurate diagnosis. Since most of the patients who receive appropriate antifungal therapy recover, it is important to resort to biopsy if a definitive diagnosis is not readily available.
